# Supplementary material for: Association between oral microbiome and seven types of cancers in East Asian population: a two-sample Mendelian randomization analysis
Source: Front Mol Biosci. 2023 Nov 21;10:1327893. doi: 10.3389/fmolb.2023.1327893 (PMC10702768; doi:10.3389/fmolb.2023.1327893)
Supplement: Supplementary file 1 [file DataSheet1.ZIP › Supplementary Materials/MR plots for tongue/tongue═╝/Lung cancer/pheno.3136_to_lung cancer_forest.pdf]

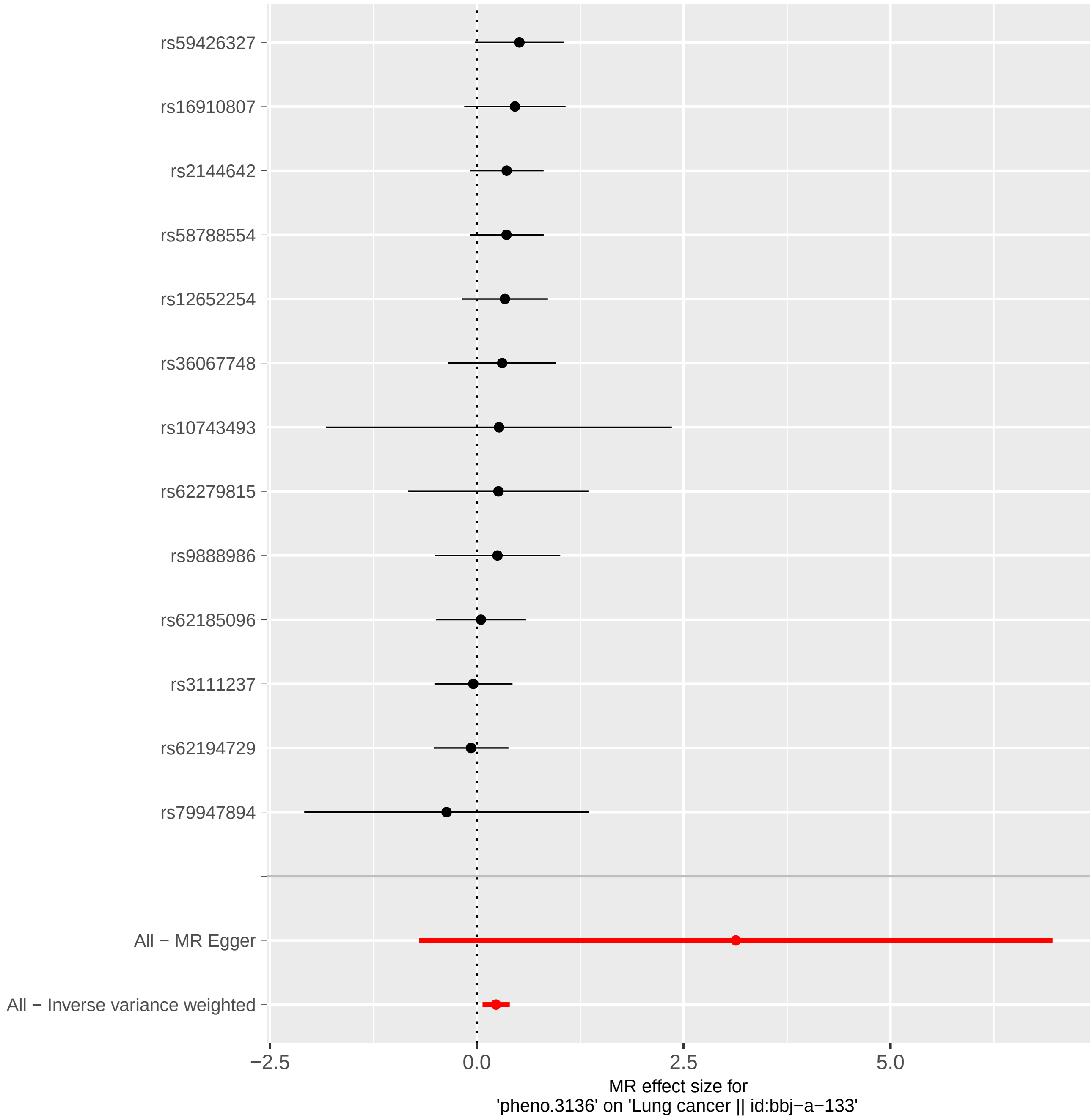
rs59426327
rs16910807
rs2144642
rs58788554
rs12652254
rs36067748
rs10743493
rs62279815
rs9888986
rs62185096
rs3111237
rs62194729
rs79947894
All − MR Egger
All − Inverse variance weighted
−2.5
0.0
2.5
5.0
MR effect size for
'pheno.3136' on 'Lung cancer || id:bbj−a−133'
